# Sorafenib or placebo in patients with newly diagnosed acute myeloid leukaemia: long-term follow-up of the randomized controlled SORAML trial

**DOI:** 10.1038/s41375-021-01148-x

**Published:** 2021-02-18

**Authors:** Christoph Röllig, Hubert Serve, Richard Noppeney, Maher Hanoun, Utz Krug, Claudia D. Baldus, Christian H. Brandts, Volker Kunzmann, Hermann Einsele, Alwin Krämer, Carsten Müller-Tidow, Kerstin Schäfer-Eckart, Andreas Neubauer, Andreas Burchert, Aristoteles Giagounidis, Stefan W. Krause, Andreas Mackensen, Walter Aulitzky, Regina Herbst, Mathias Hänel, Norbert Frickhofen, Johannes Kullmer, Ulrich Kaiser, Alexander Kiani, Hartmut Link, Thomas Geer, Albrecht Reichle, Christian Junghanß, Roland Repp, Achim Meinhardt, Heinz Dürk, Ina-Maria Klut, Martin Bornhäuser, Markus Schaich, Stefani Parmentier, Martin Görner, Christian Thiede, Malte von Bonin, Uwe Platzbecker, Johannes Schetelig, Michael Kramer, Wolfgang E. Berdel, Gerhard Ehninger, for the Study Alliance Leukaemia (SAL)

**Affiliations:** 1 Medizinische Klinik und Poliklinik I, Universitätsklinikum der Technischen Universität Dresden, Dresden, Germany; 2 Medizinische Klinik II, Universitätsklinikum Frankfurt, Frankfurt, Germany; 3 Klinik für Hämatologie und Stammzelltransplantation, Universitätsklinikum Essen, Essen, Germany; 4 Medizinische Klinik 3, Klinikum Leverkusen, Leverkusen, Germany; 5 Klinik für Innere Medizin II, Universitätsklinikum Schleswig-Holstein, Kiel, Germany; 6 Medizinische Klinik und Poliklinik II, Universitätsklinikum Würzburg, Würzburg, Germany; 7 Medizinische Klinik V, Universitätsklinikum, NCT und DKFZ Heidelberg, Heidelberg, Germany; 8 Medizinische Klinik 5, Universitätsklinik der Paracelsus Medizinischen Privatuniversität Nürnberg, Nürnberg, Germany; 9 Klinik für Hämatologie, Onkologie und Immunologie, Universitätsklinikum Gießen und Marburg and Philipps-Universität Marburg, Marburg, Germany; 10 Klinik für Onkologie, Hämatologie und Palliativmedizin, Marien Hospital Düsseldorf, Düsseldorf, Germany; 11 Medizinische Klinik 5 - Hämatologie & Internistische Onkologie, Universitätsklinikum Erlangen, Erlangen, Germany; 12 Robert-Bosch-Krankenhaus, Stuttgart, Germany; 13 Klinik für Innere Medizin III, Klinikum Chemnitz, Chemnitz, Germany; 14 Klinik Innere Medizin III, HELIOS Dr. Horst Schmidt Kliniken Wiesbaden, Wiesbaden, Germany; 15 Medizinische Klinik II, DIAKO Bremen, Bremen, Germany; 16 Medizinische Klinik II, St Bernward Krankenhaus, Hildesheim, Germany; 17 Klinik für Onkologie und Hämatologie, Klinikum Bayreuth, Bayreuth, Germany; 18 Klinik für Innere Medizin 1, Westpfalz-Klinikum, Kaiserslautern, Germany; 19 Innere Medizin III, Diakonie-Klinikum Schwäbisch Hall, Schwäbisch Hall, Germany; 20 Klinik und Poliklinik für Innere Medizin III, Universitätsklinikum Regensburg, Regensburg, Germany; 21 Medizinische Klinik III, Universitätsmedizin Rostock, Rostock, Germany; 22 2. Medizinische Klinik V, Städtisches Krankenhaus Kiel, Kiel, Germany; 23 Klinik für Hämatologie und Onkologie – Stammzelltransplantation, Agaplesion Diakonieklinikum Rotenburg, Rotenburg, Germany; 24 Klinik Hämatologie und Onkologie, St. Marien-Hospital Hamm, Hamm, Germany; 25 Klinikapotheke Universitätsklinikum TU Dresden, Dresden, Germany; 26 Klinik für Hämatologie, Onkologie und Palliativmedizin, Rems-Murr-Klinikum, Winnenden, Germany; 27 Klinik für Hämatologie, Onkologie und Palliativmedizin, Klinikum Bielefeld Mitte, Bielefeld, Germany; 28 Medizinische Klinik und Poliklinik I, Hämatologie und Zelltherapie, Universitätsklinikum Leipzig, Leipzig, Germany; 29 Medizinische Klinik A, Universitätsklinikum Münster, Münster, Germany

**Keywords:** Diseases, Acute myeloid leukaemia

## Abstract

Early results of the randomized placebo-controlled SORAML trial showed that, in patients with newly diagnosed acute myeloid leukaemia (AML), sorafenib led to a significant improvement in event-free (EFS) and relapse-free survival (RFS). In order to describe second-line treatments and their implications on overall survival (OS), we performed a study after a median follow-up time of 78 months. Newly diagnosed fit AML patients aged ≤60 years received sorafenib (*n* = 134) or placebo (*n* = 133) in addition to standard chemotherapy and as maintenance treatment. The 5-year EFS was 41 versus 27% (HR 0.68; *p* = 0.011) and 5-year RFS was 53 versus 36% (HR 0.64; *p* = 0.035). Allogeneic stem cell transplantation (allo SCT) was performed in 88% of the relapsed patients. Four years after salvage allo SCT, the cumulative incidence of relapse was 54 versus 35%, and OS was 32 versus 50%. The 5-year OS from randomization in all study patients was 61 versus 53% (HR 0.82; *p* = 0.282). In conclusion, the addition of sorafenib to chemotherapy led to a significant prolongation of EFS and RFS. Although the OS benefit did not reach statistical significance, these results confirm the antileukaemic activity of sorafenib.

## Introduction

Sorafenib is a first-generation type-II multi-kinase inhibitor with preclinical efficacy against RAS/RAF, c-KIT, vascular endothelial growth factor (VEGF) receptor, platelet-derived growth factor (PDGF) receptor kinases and FLT3 [1–4]. Due to preclinical [5] and non-randomized signals for clinical efficacy of the drug in acute myeloid leukaemia (AML), the Study Alliance Leukaemia (SAL) study group set up a randomized trial to evaluate the efficacy and tolerability of sorafenib in addition to standard therapy in primary treatment for adult AML patients up to the age of 60 years. In the first study analysis after a follow-up time of 36 months, the addition of sorafenib led to a significant prolongation of event-free (EFS) and relapse-free survival (RFS) and a trend for longer overall survival (OS) [6]. The relative risk for grade ≥3 fever, diarrhoea, bleeding, cardiac and skin events was significantly increased after sorafenib intake.

The purpose of the present study was to obtain additional data on the clinical course of patients in order to investigate the type and efficacy of second-line treatment in relapsed patients, including rates and modalities of allogeneic stem cell transplantation (allo SCT), cumulative incidence of relapse (CIR) from second complete remission (CR), and survival from first relapse. The information was used to study their influence of these parameters on OS after a prolonged follow-up time.

## Subjects and methods

### Study design and treatment

SORAML was a randomized placebo controlled double blind trial conducted in Germany. Detailed information was published previously [6]. Briefly, patients aged 18–60 years with newly diagnosed AML irrespective of the FLT3 mutational status with an Eastern Cooperative Oncology Group performance score ≤2 and adequate cardiac, renal and liver function were eligible for inclusion in the trial. After enrolment, patients were randomized 1:1 by central block randomization with allocation concealment to placebo or sorafenib treatment. Randomization was performed in six strata (favourable or high risk and four intermediate risk strata according to NPM1/FLT3-ITD status) by the SAL data centre using randomization sequences with variable block length generated by an R program for each stratum. Patients, treating physicians, study investigators assessing outcomes and statisticians analysing the data were blinded to study arm assignment. Patients received the first cycle of induction treatment consisting of cytarabine 100 mg/m^2^ per day as a continuous infusion for 7 days plus daunorubicin 60 mg/m^2^ as short infusion on days 3–5 (“DA 7 + 3”), and patients received either two capsules of sorafenib 200 mg or sorafenib matching placebo twice daily on days 10–19. Responding patients were scheduled for a second identical induction from day 22, whereas patients with no response were treated with high-dose cytarabine as 3-h infusion 3 g/m^2^ twice daily on days 1–3 plus mitoxantrone short infusion 10 mg/m^2^ on days 3–5 (“HAM”), both followed by sorafenib or placebo on days 10–19. After induction, intermediate-risk patients with a family donor and adverse-risk patients with a matched donor were offered SCT, whereas all other patients proceeded to three cycles of cytarabine-based consolidation with cytarabine as 3-h infusion 3 g/m^2^ twice daily on days 1, 3, and 5 followed by study medication from day 8 until 3 days before the next consolidation cycle. For maintenance therapy, sorafenib at a dose of 400 mg twice daily or placebo was administered continuously for 12 months after the last consolidation cycle. Patients who received an allo SCT stopped study treatment by the commencement of conditioning and did not receive sorafenib maintenance after SCT. The trial design is summarized according to CONSORT statement in Supplemental Fig. SF1.

### Endpoints and sample size

The primary outcome was EFS, with an event being either primary treatment failure or relapse or death. For the determination of primary induction failure, all patients were included who had not achieved a CR or CRi on day 35 after the completion of double induction either with DA + DA or DA + HAM according to the study protocol. In patients who received at least one consolidation cycle, the interval for CR/CRi demonstration was extended from day 35 after double induction until the beginning of the first consolidation cycle.

The sample size calculation for the trial was based on the assumption that sorafenib would prolong the primary endpoint EFS from 9 to 13.5 months. In order to reject the null hypothesis of no difference between the two study arms with 80% power at a significance level of 0.05 in a two-sided stratified log-rank test, a sample size of 276 patients and approximately 191 events were required. Because of one pre-planned interim analysis after 95 events, the significance level for the final analysis was adjusted to 0.046.

Secondary endpoints were RFS, OS, CR rate and toxicity (incidence of adverse events (AEs) ≥ grade 3). Remission status after two induction cycles was centrally reviewed.

### Statistical analyses

All randomized patients who received at least one dose of study medication formed the full analysis set for the intention to treat analyses. Patients were not censored at the time of allo SCT. Standard statistical methods were used for descriptive analyses. The Kaplan–Meier method and a stratified log-rank test for accounting for stratified randomization were used for unadjusted analyses of survival endpoints. Secondary multivariable analyses for survival outcomes were done using Cox regression models with likelihood-ratio tests for significance, adjusting for the influence of established prognostic parameters. Due to the longer follow-up, early censored observations were updated leading to slightly improved estimates compared to the earlier published results. Analyses were performed using SPSS version 20.0.0.1 and the software R version 3.1.1.

Informed consent was obtained from all patients according to the Declaration of Helsinki; the trial was approved by the responsible Ethics Committees of all participating sites, overseen by a data monitoring committee and registered on clinicaltrials.gov (NCT00893373) and in the EU Clinical Trials Register (2008-004968-40).

## Results

### Patient disposition and response

Between 27 March 2009 and 28 November 2011, 276 patients were enrolled in 25 German study sites. Nine patients did not receive study medication (3 withdrawals of consent, 3 AEs, 1 secondary malignancy, 2 diagnosis changes) and were therefore excluded from the analyses; thus a total number of 267 patients formed the full analysis set for the intention-to-treat analyses (134 sorafenib and 133 placebo). A total number of 46/267 patients had FLT3-internal tandem duplication (ITD)-positive AML (17%) and 86/267 had NPM1-mutated AML (33%, Table 1). In 196 patients with available samples for subsequent analyses, 7 were FLT3-TKD mutated (4%).

**Table 1:** Patient characteristics.

Demographics	Placebo	Sorafenib
	*n* = 133	*n* = 134
Age (years), median [min, max]	50 [19, 60]	50 [20, 60]
Female, *n* (%)	70 (52.6)	63 (47.0)
Secondary AML, *n* (%)	20 (15.0)	14 (10.5)
ECOG status, *n* (%)		
ECOG 0	48 (36.1)	42 (31.3)
ECOG 1	70 (52.6)	82 (61.2)
ECOG 2	1 (0.8)	—
ECOG missing	14 (10.5)	10 (7.5)
Bone marrow blasts in %, median [min, max]	62 [20, 96]	64 [20, 100]
Missing, *n* (%)	6 (4.5)	3 (2.2)
White blood count in Gpt/l, median [min, max]	8.8 [0.1, 187.8]	8.8 [0.1, 277.8]
Platelet count in Gpt/l, median [min, max]	57 [1, 554]	59 [1, 291]
Lactate dehydrogenase in U/l, median [min, max]	364 [133, 871]	335 [87, 984]
Missing, *n* (%)	17 (12.8)	15 (11.2)
Cytogenetic risk group, *n* (%)		
Low risk (LR)	11 (8.3)	14 (10.4)
Intermediate risk (IR)	89 (66.9)	88 (65.7)
High risk (HR)	26 (19.5)	23 (17.2)
Could not be assessed	7 (5.2)	9 (6.7)
Normal karyotype, *n* (%)	66 (49.6)	68 (50.7)
Stratification, *n* (%)		
HR cytogenetics	26 (19.5)	23 (17.2)
LR cytogenetics	11 (8.3)	14 (10.4)
IR cytogenetics, NPM mut, FLT3-ITD wt	30 (22.6)	30 (22.3)
IR cytogenetics, NPM mut, FLT3-ITD mut	14 (10.5)	13 (9.7)
IR cytogenetics, NPM wt, FLT3-ITD mut	5 (3.8)	7 (5.2)
IR cytogenetics, NPM wt, FLT3-ITD wt	47 (35.3)	47 (35.1)
NPM1 mutation, *n* (%)	43 (32.3)	43 (32.1)
Missing, *n* (%)	1 (0.8)	3 (2.2)
FLT3-ITD mutation, *n* (%)	23 (17.3)	23 (17.2)
Missing, *n* (%)	—	1 (0.8)
FLT3-ITD/wt ratio, median [min, max]	0.49 [0.01, 1.53]	0.47 [0.06, 14.3]

Aberrations t(8;21), inv(16) and t(16;16) were considered favourable risk; −7, −5, −5q, inv(3), t(3;3), t(6;9), t(6;11), t(11;19) and ≥3 aberrations were categorised as high risk, whereas normal karyotype and all other aberrations were considered as intermediate risk.

The CR rate was 60% (81/134) in the sorafenib arm and 59% (78/133) in the placebo arm. More sorafenib patients discontinued study treatment after induction I, mainly due to AEs and withdrawal of consent. Due to variations in donor availability, physical condition, treatment response and patients’ preference, not all patients with intermediate and high cytogenetic risk were transplanted: 42/134 patients (31%) in the sorafenib arm and 35/133 patients (26%) in the placebo arm received an allo SCT in first CR. Cytarabine consolidation was started in 42% (56/133) of sorafenib patients and 49% (65/133) of placebo patients followed by maintenance in 25% (33/134) versus 32% (43/133) of patients in the sorafenib and placebo arms, respectively. The median duration of treatment was 37.5 days in the sorafenib arm and 41 days in the placebo arm (*p* = 0.13). The median duration of treatment in all patients who did not receive an allo SCT as first-line treatment was 63 days in the sorafenib arm and 111.5 days in the placebo arm (*p* = 0.15). In patients who started at least one cycle of HIDAC consolidation, the median duration of treatment was 248.5 days in the sorafenib arm and 283 days in the placebo arm (*p* = 0.66). Induction mortality on study was 3% (4/134) versus 1.5% (2/133) in the sorafenib and placebo arms, respectively (Supplemental Fig. SF2).

### Event-free survival

**Fig. 1:**
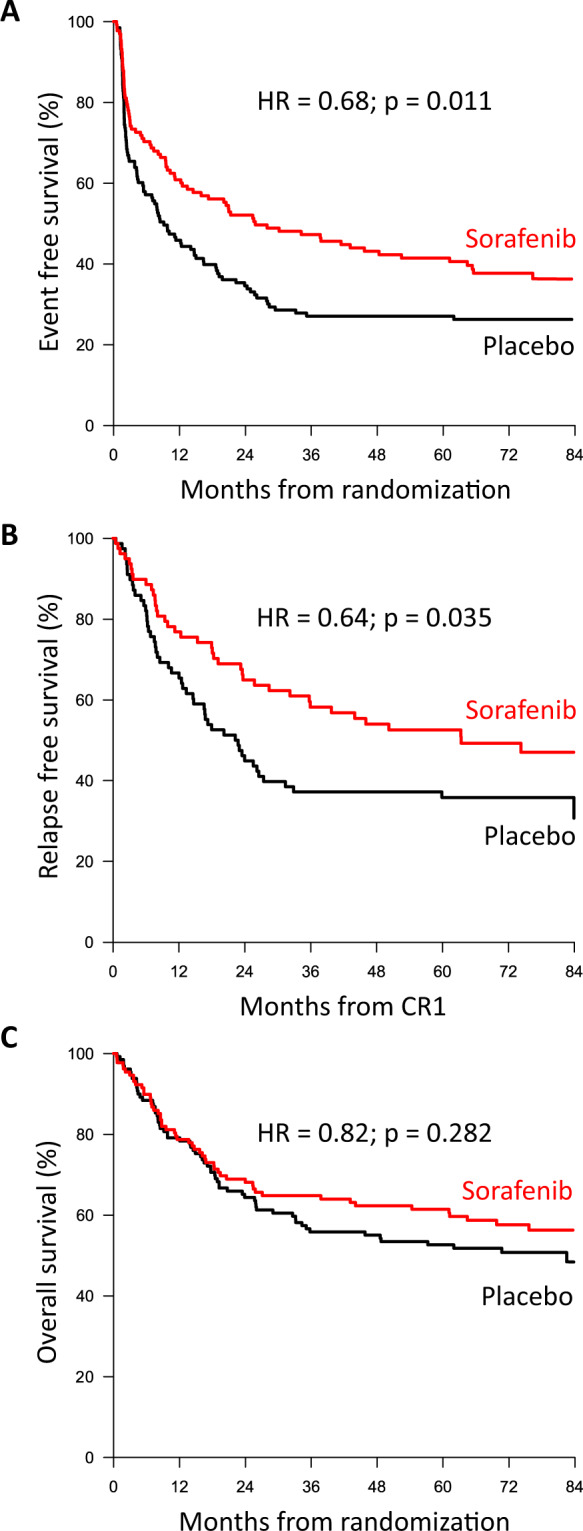
Survival outcomes based on randomization to the sorafenib arm (red line) or placebo arm (black line). Event-free survival (**A**), relapse-free survival (**B**) and overall survival (**C**) with no censoring for allogeneic stem cell transplantation.

After a median follow-up of 78 months, 179 events had occurred, 80 in the sorafenib arm and 99 in the placebo arm. The 5-year EFS in the two arms were 41% (95% confidence interval (CI) 34–51) versus 27% (95% CI 21–36) with an unadjusted hazard ratio (HR) of 0.68 (95% CI 0.51–0.91; *p* = 0.011; Fig. 1). In a multivariable Cox model accounting for the established prognostic parameters age, cytogenetic risk, NPM1 and FLT3-ITD mutation status, lactate dehydrogenase (LDH), white blood cell (WBC) and secondary AML, treatment with sorafenib retained its significant influence on EFS with an adjusted HR of 0.61 (95% CI 0.44–0.87; *p* = 0.006). Favourable cytogenetics and NPM1 mutation were associated with superior EFS, whereas adverse cytogenetics conveyed a negative prognosis (Table 2). Subgroup analyses revealed that patients with FLT3-ITD benefitted more from sorafenib than patients without FLT3-ITD, corresponding to a HR of 0.55 (95% CI 0.28–1.08) versus 0.71, (95% CI 0.51–0.99; see Supplemental Table ST1 and Supplemental Figs. SF3 and SF4). If patients with FLT3-ITD mutations were excluded from the study population, the increases in EFS and RFS in the sorafenib group remained significant. Across the four possible FLT3-ITD-NPM1 strata, the largest benefit was observed in FLT3-ITD-NPM1wt patients (HR 0.36, see Supplemental Fig. SF5).

**Table 2:** Hazard ratios for EFS, RFS and OS according to the multivariable Cox regression models.

	Event-free survival			Relapse-free survival			Overall survival		
	Hazard ratio	95% CI	*p*	Hazard ratio	95% CI	*p*	Hazard ratio	95% CI	*p*
Sorafenib versus placebo	0.614	0.435–0.867	0.006	0.570	0.354–0.917	0.021	0.742	0.490–1.124	0.160
Age	1.014	0.997–1.031	0.120	1.022	0.997–1.047	0.080	1.033	1.010–1.058	0.006
ELN risk favourable	0.481	0.245–0.944	0.033	0.598	0.259–1.383	0.229	0.344	0.132–0.899	0.030
ELN risk adverse	1.801	1.137–2.855	0.012	1.913	0.861–4.254	0.112	1.989	1.191–3.322	0.009
NPM1	0.373	0.224–0.621	<0.001	0.513	0.268–0.980	0.044	0.381	0.198–0.735	0.004
FLT3-ITD	0.532	0.254–1.116	0.095	1.499	0.529–4.246	0.446	0.801	0.374–1.718	0.570
NPM1 × FLT3-ITD interaction	5.715	2.068–15.80	0.001	2.811	0.737–10.72	0.130	3.782	1.124–12.72	0.032
Log10 of LDH	1.609	0.619–4.183	0.328	1.024	0.251–4.174	0.973	3.174	1.060–9.501	0.039
Log10 of WBC	1.040	0.741–1.458	0.822	1.135	0.699–1.843	0.607	0.787	0.527–1.175	0.241
sAML	0.607	0.334–1.104	0.102	0.368	0.121–1.113	0.076	0.547	0.259–1.155	0.113
tAML	0.734	0.295–1.827	0.506	1.122	0.385–3.268	0.833	1.106	0.398–3.074	0.847

### Relapse-free survival and relapse from CR

In the 159 patients with a CR, 5-year RFS was 53% in the sorafenib arm and 36% in the placebo arm, with an unadjusted HR of 0.64 (95% CI 0.42–0.97; *p* = 0.035, Fig. 1). Multivariable analyses indicated a HR of 0.57 (95% CI 0.35–0.92, *p* = 0.021) after adjustment for other prognostic variables like age, cytogenetic risk, NPM1 and FLT3-ITD mutation status, LDH, WBC and secondary AML (Table 2, Supplemental Table ST1 and Supplemental Figs. SF3 and SF4). In patients who did not receive an allo SCT as postremission treatment in first CR, a clear benefit in RFS did not translate into an OS benefit, most likely due to the fact that, in relapse, the majority of patients was rescued with an allo SCT, thereby reducing the effect of first-line treatment. In patients with an allo SCT in first CR, there was a clear OS benefit (Supplemental Figs. SF6 and SF7).

**Fig. 2:**
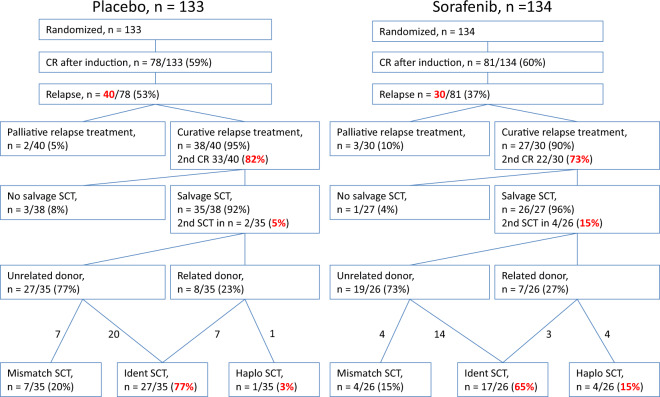
Treatment of patients after first relapse outside the SORAML trial. CR complete remission, OS overall survival, SCT allogeneic stem cell transplantation, ident HLA-identical donor (10/10 matching alleles), haplo haploidentical donor (5/10 matching alleles).

The number of relapses from CR was higher in the placebo arm. Forty relapses occurred in the placebo arm and 30 in the sorafenib arm. The CIR after 5 years was 36% (95% CI 25–47) and 50% (95% CI 39–61) in the sorafenib and placebo arm, respectively (*p* = 0.087). Figure 2 and Supplemental Table ST2 show the treatment after first relapse.

Among patients relapsing after placebo pretreatment, 95% received intensive curative salvage therapy as opposed to 90% of patients after sorafenib pretreatment. Among intensively treated patients, a lower number achieved a second CR after first-line treatment with sorafenib than after placebo (73% versus 82%; *p* = 0.528). Among all the relapsed cases, 87 and 88% received an allo SCT as part of relapse treatment after previous sorafenib or placebo in first-line treatment, respectively. Although about the same percentage of patients were transplanted after salvage therapy, the fraction of second transplants (15% versus 5%) and also the proportion of haploidentical allo SCTs (15% versus 3%) was higher in sorafenib patients. The percentage of patients transplanted after salvage treatment who still had active disease was 57% in the sorafenib versus 47% in the placebo patients.

**Fig. 3:**
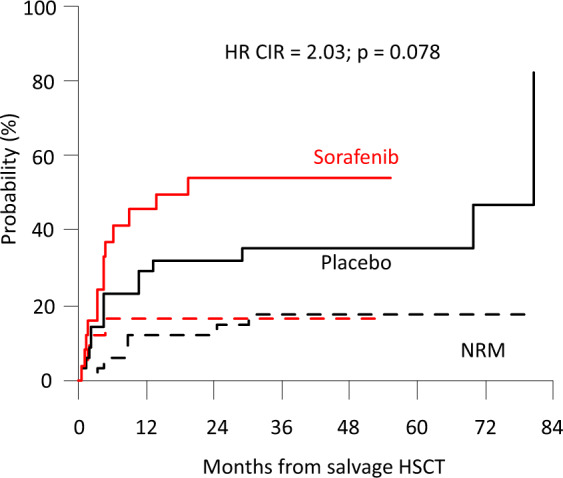
Outcomes after salvage allo SCT after pretreatment with either sorafenib (red lines) or placebo (black lines) during first-line treatment. Cumulative incidence of relapse (CIR, continuous lines) and non-relapse mortality (NRM, dashed lines).

**Fig. 4:**
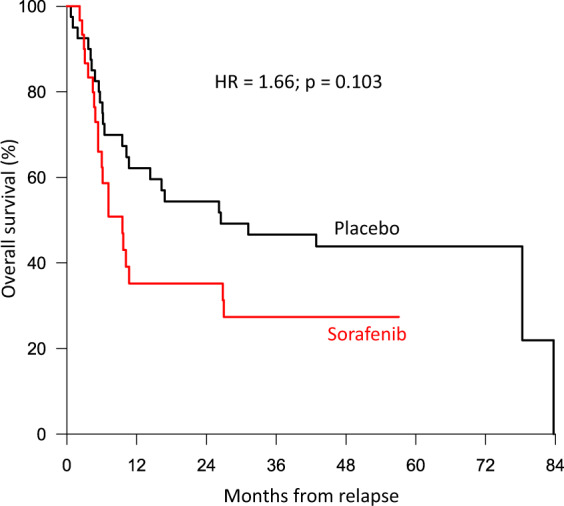
Overall survival (OS) from the time of relapse. Outcome for patients with pretreatment in the sorafenib arm (red line) or placebo arm (black line).

In the analysis of relapse incidence and non-relapse mortality (NRM) from salvage transplant, we observed that differences in the frequency of second transplant and haploidentical SCT did not affect NRM. However, sorafenib patients had a higher risk of relapse with a 4-year CIR from allo SCT of 54 versus 35% (Fig. 3) and a 4-year OS from allo SCT of 32 versus 50% after first-line treatment with sorafenib versus placebo, respectively. The median duration of CR2 from salvage allo SCT was 19 months in sorafenib-pretreated patients and 70 months in placebo-pretreated patients. This may partly be because fewer patients were transplanted in second CR. The 2-year OS from the time of relapse was shorter for patients relapsing after sorafenib with 35 versus 54% after placebo (*p* = 0.103, Fig. 4). Most likely, this is due to (i) less curative salvage treatments, (ii) lower CR rates after salvage, and therefore (iii) a higher risk of relapse or progression. Relapsing patients pretreated with sorafenib did not display more adverse baseline characteristics than placebo-treated patients (Supplemental Table ST3), and the median duration of the first CR was longer in the sorafenib group (17 versus 11 months).

The overall NRM in all randomized patients after 5 years was 12% (95% CI 6–18) and 14% (95% CI 7–20) in the sorafenib and placebo arms, respectively (*p* = 0.768).

### Overall survival

After 5 years from randomization, median OS was not reached in either arm; 61% (95% CI 53–71) and 53% (95% CI 45–62) of patients were alive in the sorafenib and placebo arm, respectively; the unadjusted HR was 0.82 (95% CI 0.57–1.18; *p* = 0.282, Fig. 3). The HR for death in the sorafenib arm in a multivariable Cox model accounting for other prognostic parameters was 0.74 (95% CI 0.49–1.12, *p* = 0.16, Table 2). For all survival outcomes EFS, RFS and OS, the risk reduction by sorafenib was larger in the subgroup of FLT3-ITD patients. These differences were not statistically significant (Supplemental Table ST1 and Supplemental Figs. SF3 and SF4).

## Discussion

The first analysis of the randomized, placebo-controlled SORAML trial after a median follow-up time of 3 years demonstrated the feasibility of adding sorafenib to intensive standard chemotherapy in younger AML patients and a significant antileukaemic efficacy in terms of significantly prolonged EFS and RFS and a trend for longer OS in patients receiving sorafenib instead of placebo. Here we present results after prolonged follow-up of 78 months. No new safety signals were observed, and the cumulative incidence of NRM remained stable after 3 years and showed no significant differences between the two study arms.

The mature survival data confirm a significant prolongation of EFS by addition of sorafenib to standard chemotherapy. Since the CR rate, the early death rate and the number of deaths in CR were similar in both treatment arms, this effect is mainly attributable to a reduction of relapses and a prolongation of the time in CR. This is confirmed by a significant RFS prolongation with a HR of 0.64, indicating that a number of 5–6 patients need to be treated in order to prevent one relapse or death. EFS and RFS prolongation are significant and clinically relevant since salvage treatment with or without allo SCT could be prevented or significantly delayed by sorafenib treatment. Possible mechanisms for the antileukaemic activity of sorafenib are most likely the inhibition of kinases in the RAF pathway [5], c-KIT and FLT3. Interestingly, the beneficial effect was not restricted to the FLT3-ITD subgroup and was detectable even when FLT3-ITD-negative patients were analysed separately. Additional effects of sorafenib might be explained by its anti-angiogenic activity through the inhibition of tyrosine kinase function of pro-angiogenic receptors such as VEGF of PDGF receptors may contribute to the antileukaemic activity [7].

Five years after randomization, median OS was not reached in either arm, with a difference of 8% favouring the sorafenib group and corresponding to an unadjusted HR of 0.82 and 0.74 after accounting for imbalances in prognostic variables. This difference did not reach statistical significance. In order to explore why RFS did not translate into a significant OS prolongation, we gathered data on relapse treatment modality and efficacy. Whereas treatment modalities such as the proportion of palliative versus intensive treatment and rates of allo SCT did not differ between the two study arms, there was a lower rate of second CRs and a higher incidence of relapse after salvage allo SCT in patients who received sorafenib. This indicates that salvage treatment in patients relapsing after sorafenib treatment was slightly less effective and durable than after placebo treatment and pointing towards more resistant disease compared with patients with no previous sorafenib exposure. This constellation most likely explains the observed trend for shorter OS from relapse in sorafenib-pretreated patients and the lower beneficial effect of sorafenib on survival in the study population. Clinical characteristics at baseline or duration of first CR do not explain the adverse clinical course of relapsed patients pretreated with sorafenib. Since no relapse samples were collected as part of the trial, a study limitation is the lack of molecular findings or preclinical data explaining the resistance of post-sorafenib relapses.

Midostaurin, another first-generation tyrosine kinase inhibitor (TKI) has been explored in first-line treatment of fit younger AML patients. The RATIFY trial had a similar study design but enrolled exclusively patients with FLT3 mutations. As opposed to SORAML, not only EFS and RFS but also OS was significantly prolonged by midostaurin. With a HR for OS of 0.78 [8], the risk reduction for death in the RATIFY trial was in a similar range as in SORAML; however, with a much higher patient number, results were statistically significant. Whereas both TKIs are multikinase inhibitors, the type-I inhibitor midostaurin also acts against FLT3 with point mutations (TKD). The prevalence of FLT3-TKD in the RATIFY trial was higher than in an unselected AML population (23% versus 5–10%) [9, 10], and midostaurin had the highest efficacy in this subgroup (HR 0.65). Although small in size, results of subgroup analyses indicate a stronger beneficial effect of sorafenib in FLT3-ITD-mutated AML patients. The HR for OS in FLT3-ITD patients in SORAML was 0.55 as opposed to 0.80 in the RATIFY trial. However, in SORAML, the significant EFS and RFS prolongation was still detectable after the 46 FLT3-ITD patients were removed from the analysis set, indicating beneficial effects also in patients without this mutation (data not shown). The selection of FLT3-mutated patients, the inhibitory effect on FLT3-TKD and the higher number of enrolled patients allowing a greater power to detect a significant difference in OS may be the most likely reasons for the significant OS benefit in the RATIFY, which lead to regulatory approval of midostaurin for use in FLT3-mutated AML.

More recently, evidence has evolved showing that multikinase inhibition may not be the only mode of action for sorafenib. Based on preclinical data, the drug is able to increase the immunogenicity of leukaemia cells via induction of interleukin-15 production, thereby enhancing T cell activation [11]. This mechanism may be of particular relevance in the post-allo SCT setting, fostering the graft-versus-leukaemia effect. Randomized proof of this concept comes from the SORMAIN trial using pre-emptive sorafenib versus placebo in FLT3-ITD patients for 2 years after allo SCT. Although prematurely closed for slow recruitment, the trial showed a significant prolongation of 2-year RFS from 53 to 85% (HR 0.39; *p* = 0.0135) and also of OS [12].

The presented results contribute to the body of evidence for the antileukaemic activity of sorafenib: first-line in combination with intensive cytarabine-based chemotherapy [13], in combination with azacitidine as first-line [14] or relapse treatment [15, 16], or as single agent after allo SCT [17]. The SORAML trial represented the first randomized trial demonstrating clinically meaningful antileukaemic activity of a TKI in first-line treatment of AML.

In summary, the addition of sorafenib to standard intensive treatment led to a significant EFS and RFS prolongation after prolonged follow-up. This effect was less pronounced for OS and did not reach statistical significance. For future practice, these results do not support standard use of sorafenib in intensive first-line treatment, but the demonstrated antileukaemic efficacy of sorafenib may justify its use off label as maintenance after allo SCT or in combination with azacitidine or cytarabine in the relapsed setting.

## Supplementary information

Supplemental Material
