# Supplementary material for: Sorafenib or placebo in patients with newly diagnosed acute myeloid leukaemia: long-term follow-up of the randomized controlled SORAML trial
Source: Leukemia. 2021 Feb 18;35(9):2517–25. doi: 10.1038/s41375-021-01148-x (PMC8410595; doi:10.1038/s41375-021-01148-x)

Supplemental Material for Publication

Sorafenib or Placebo in Patients with Newly Diagnosed Acute Myeloid Leukemia: Long-Term Follow Up of the SORAML Trial

Christoph Röllig[1*], Hubert Serve[2*], Richard Noppeney[3], Maher Hanoun[3], Utz Krug[6], Claudia D Baldus[4], Christian H Brandts[2], Volker Kunzmann[5], Hermann Einsele[5], Alwin Krämer[6], Carsten Müller-Tidow[6], Kerstin Schäfer-Eckart[7], Andreas Neubauer[8], Andreas Burchert[8], Aristoteles Giagounidis[9], Stefan W Krause[10], Andreas Mackensen[10], Walter Aulitzky[11], Regina Herbst[12], Mathias Hänel[12], Norbert Frickhofen[13], Johannes Kullmer[14], Ulrich Kaiser[15], Alexander Kiani[16], Hartmut Link[17], Thomas Geer[18], Albrecht Reichle[19], Christian Junghanß[20], Roland Repp[21], Achim Meinhardt[22], Heinz Dürk[23], Ina-Maria Klut[24], Martin Bornhäuser[1], Markus Schaich[25], Stefani Parmentier[25], Martin Görner[26], Christian Thiede[1], Malte von Bonin[1], Uwe Platzbecker[27], Johannes Schetelig[1], Michael Kramer[1], Wolfgang E Berdel[28*], and Gerhard Ehninger[1*] for the Study Alliance Leukaemia (SAL)

The the full trial protocol can be accessed under

https://www.sal-aml.org/sal/studien/abgeschlossene-studien/soraml-1

Supplemental Table ST1. 5-year event-free survival, relapse-free survival and overall survival of study patients stratified according to FLT3-ITD mutation status.

	Sorafenib Arm		Placebo Arm	
	FLT3-ITD	FLT3wt/-TKD	FLT3-ITD	FLT3wt/-TKD
5-year EFS in % (95%-CI)	34.8 (19.9 – 60.9)	43.3 (34.7 – 54.1)	8.7 (2.3 – 32.7)	30.9 (23.4 – 40.9)
5-year RFS in % (95%-CI)	42.9 (26.2 – 70.2)	54.5 (45.0 – 66.0)	22.7 (10.5 – 49.1)	44.5 (35.5 – 55.8)
5-year OS in % (95%-CI)	59.7 (42.4 – 84.0)	62.4 (53.6 – 72.6)	39.1 (23.5 – 65.1)	55.6 (46.8 – 65.9)

Supplemental Table ST2. 5-year event-free survival, relapse-free survival and overall survival of patients relapsed after study treatment, stratified according to FLT3-ITD mutation status.

n/% (95%-CI)	Sorafenib Arm	Placebo Arm
Relapse	30/81<br>37% (27 – 48%)	40/78<br>51% (40-63%)
Relapse treatment		
- Palliative	3/30<br>10% (2-27%)	2/40<br>5% (1-17%)
- Curative	27/30<br>90% (73-98%)	38/40<br>95% (83-99%)
Second CR<br>(after curative salvage)	22/30<br>73% (54-88%)	33/40<br>82% (67-93%)
Salvage SCT	26/30<br>87% (69-96%)	35/40<br>88% (73-96%)
- Second SCT	4/30<br>13% (4-31%)	2/40<br>5% (1-17%)
Relapse after salvage SCT	13/26<br>50% (30-70%)	14/35<br>40% (24-58%)

Supplemental Table ST3. Patient characteristics. Aberrations t(8;21), inv(16) and t(16;16) were considered favourable risk; -7, -5, -5q, inv(3), t(3;3), t(6;9), t(6;11), t(11;19) and ≥3 aberrations were categorized as high risk, whereas normal karyotype and all other aberrations were considered intermediate risk

**Demographics**		**Placebo** n = 40	**Sorafenib** n = 30
Age (years), median [min, max]		49 [26, 60]	48 [20, 60]
Female, n (%)		16 (40·0)	12 (40·0)
Secondary AML, n (%)		4 (10·0)	3 (10·0)
ECOG status, n (%)			
	ECOG 0	15 (37·5)	8 (26·7)
	ECOG 1	19 (47·5)	20 (66·7)
	ECOG 2	-	-
	ECOG missing	6 (15·0)	2 (6·7)
Bone marrow blasts in %, median [min, max]		75 [24, 96]	67 [35, 90]
	missing, n (%)	1 (2·5)	1 (3·3)
White blood count in Gpt/l, median [min, max]		19·0 [0·4, 134·3]	12·8 [0·7, 277·8]
Platelet count in Gpt/l, median [min, max]		49 [7, 354]	72 [19, 291]
Lactate dehydrogenase in U/l, median [min, max]		513 [153, 859]	316 [115, 984]
	missing, n (%)	6 (15·0)	5 (16·7)
Cytogenetic risk group, n (%)			
	low risk (LR)	18 (45·0)	11 (36·7)
	intermediate risk (IR)	13 (32·5)	13 (43·3)
	high risk (HR)	7 (17·5)	5 (16·7)
	could not be assessed	2 (5·0)	1 (3·3)
Normal karyotype, n (%)		22 (55·0)	18 (60·0)
Stratification, n (%)			
	HR cytogenetics	7 (17·5)	5 (16·7)
	LR cytogenetics	5 (12·5)	3 (10·0)
	IR cytogenetics, NPM mut, FLT3-ITD wt	10 (25·0)	5 (16·7)
	IR cytogenetics, NPM mut, FLT3-ITD mut	7 (17·5)	5 (16·7)
	IR cytogenetics, NPM wt, FLT3-ITD mut	1 (2·5)	-
	IR cytogenetics, NPM wt, FLT3-ITD wt	10 (25·0)	12 (40·0)
NPM1 mutation, n (%)		18 (45·0)	11 (36·7)
	missing, n (%)	-	-
FLT3-ITD mutation, n (%)		9 (22·5)	6 (20·0)
	missing, n (%)	-	-
FLT3-ITD/wt ratio, median [min, max]		0·36 [0·07, 1·02]	0·45 [0·06, 14·3]

Supplemental Figure SF1. Trial design. AML, acute myeloid leukaemia; DA, daunorubicin plus cytarabine 7+3; HAM, high-dose cytarabine plus mitoxantrone; sora, sorafenib; allo SCT, allogeneic stem cell transplantation; HiDAC, high-dose cytarabine; favourable risk (FR): t(8;21), inv(16); high risk (HR): -7, -5, -5q, inv(3), t(3;3), t(6;9), t(6;11), t(11;19) or ≥3 aberrations or insufficient response on day 16 after DA I (in this case second induction with HAM); intermediate risk (IR): all cytogenetics not FR or HR

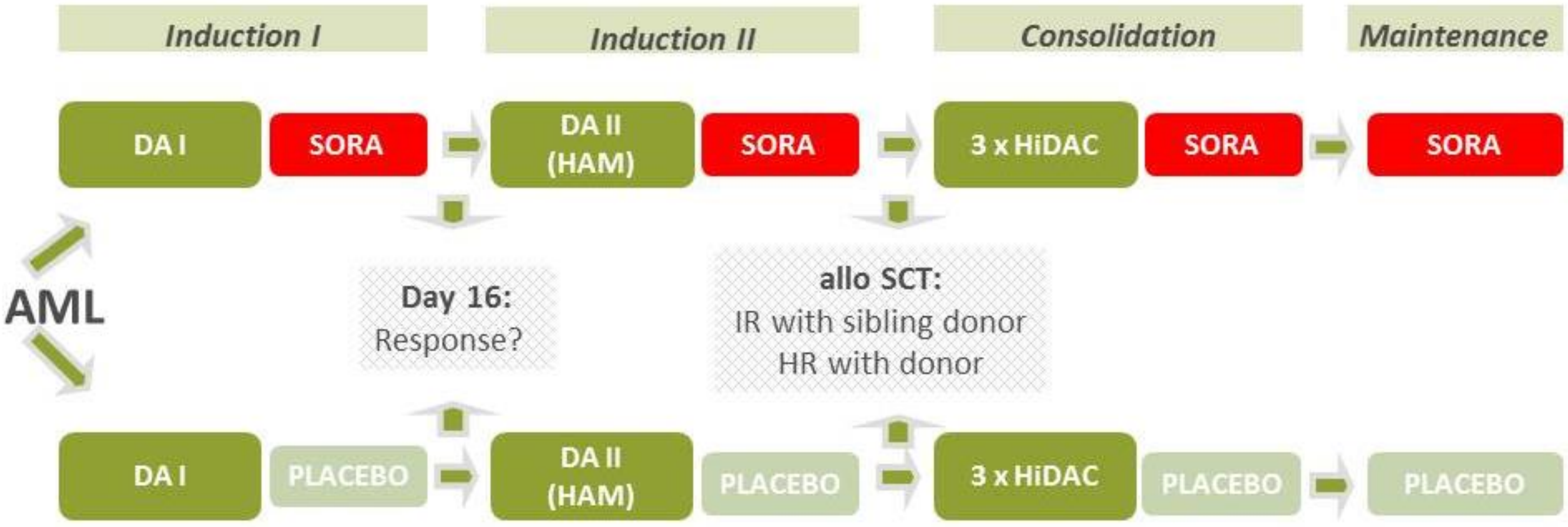

Supplemental Figure SF2. Patient flow according to the CONSORT statement. Allo SCT, allogeneic stem cell transplantation.

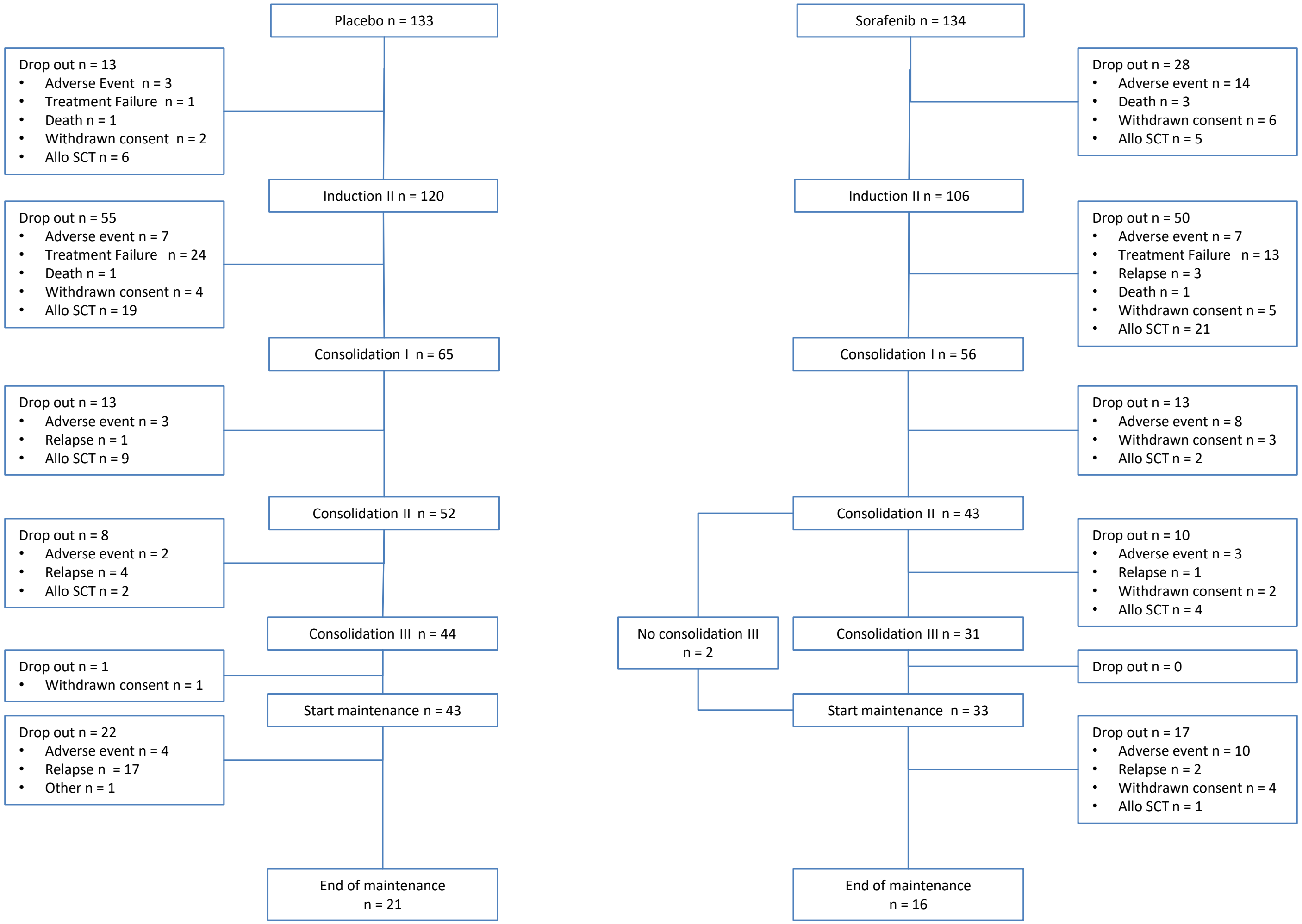

Supplemental Figure SF3. Event-free survival (A), relapse-free survival (B) and overall survival (C) in FLT3wt/-TKD patients.

A

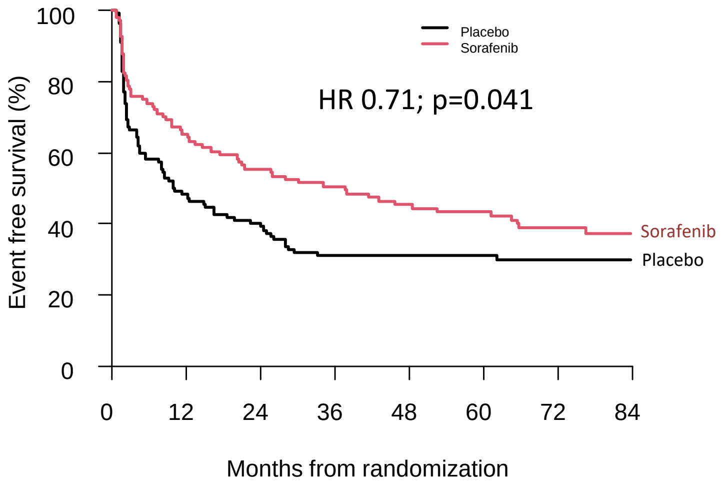

B

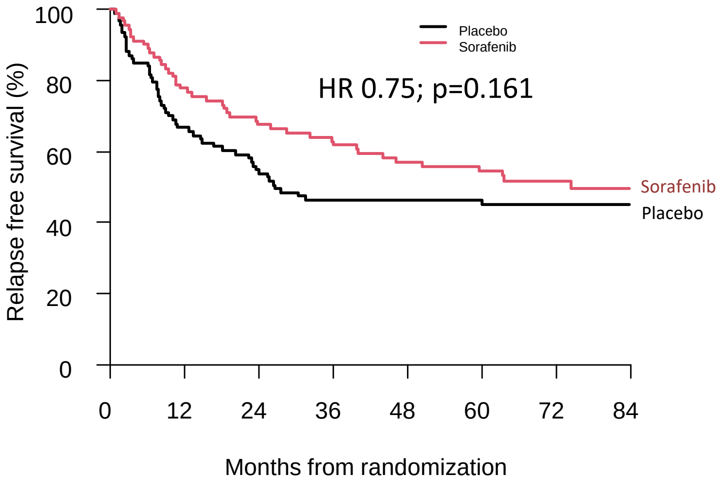

C

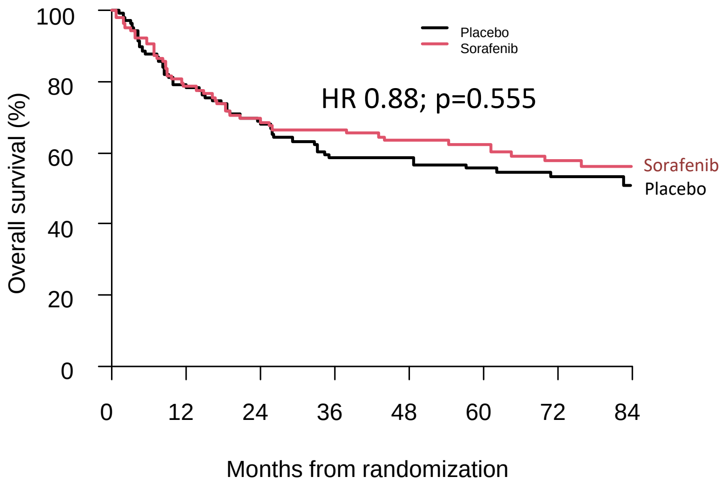

Supplemental Figure SF4. Event-free survival (A), relapse-free survival (B) and overall survival (C) in FLT3-ITD patients.

A

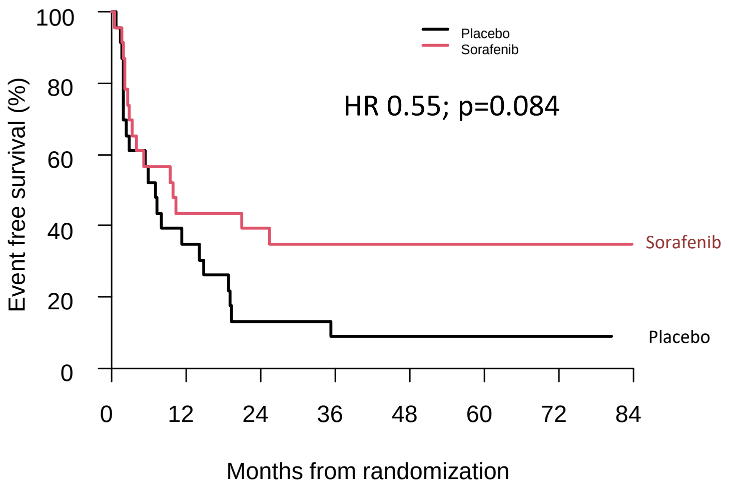

B

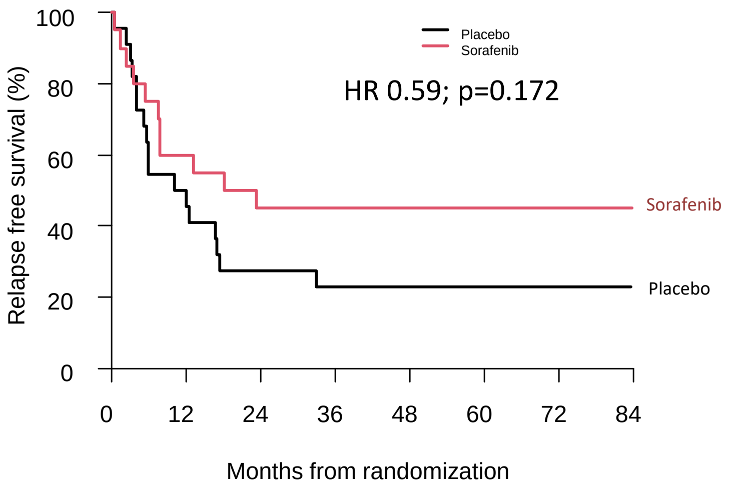

C

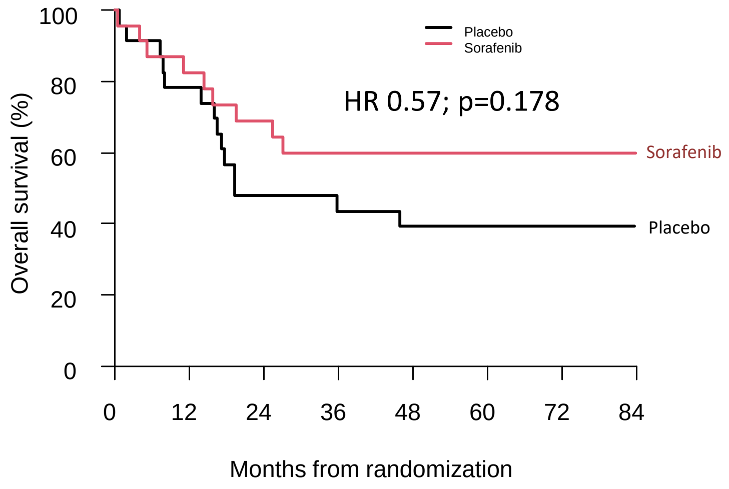

Supplemental Figure SF5. Forrest plot of sofarenib effect on EFS in patient subgroups defined by FL3-ITD and NPM1 mutational status. (A) presence of FLT3-ITD mutation; (B) FLT3-ITD-NPM1 strata.

A

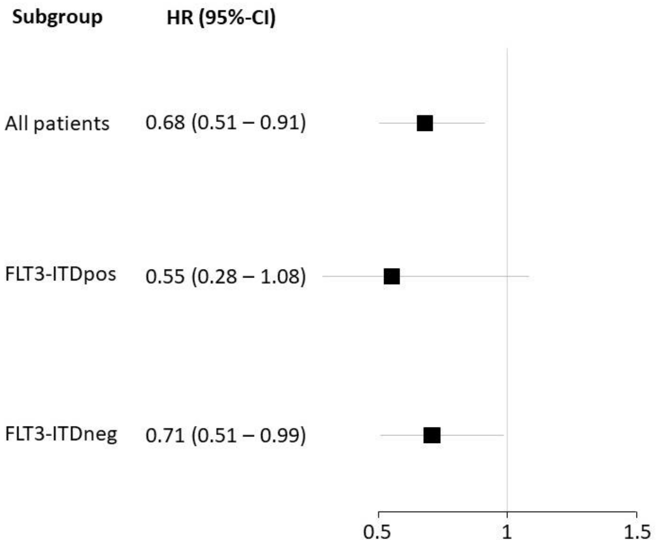

B

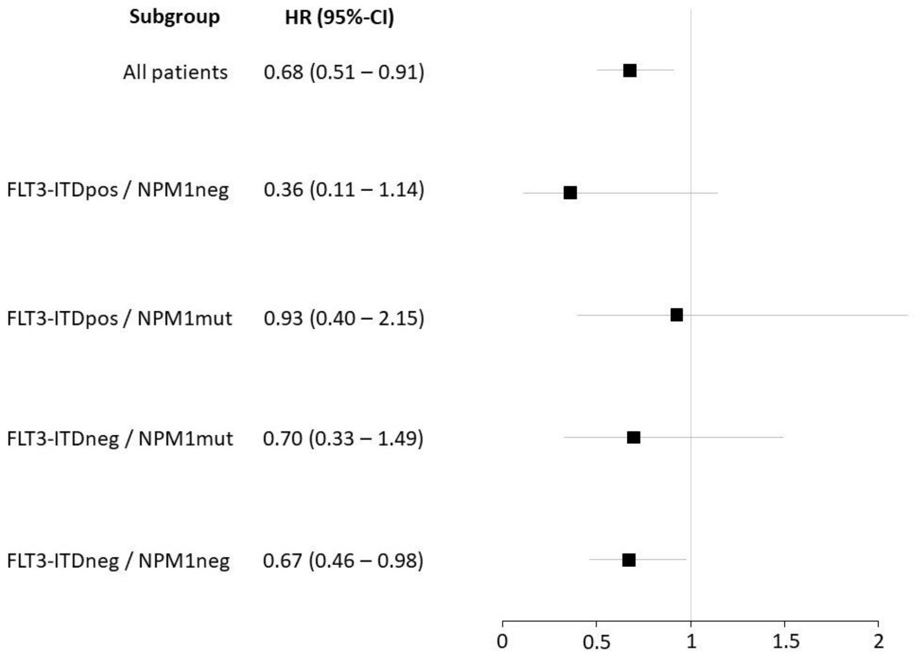

Supplemental Figure SF6. Relapse-free survival (A) and overall survival (B) censoring patients by the time of allogeneic SCT.

A

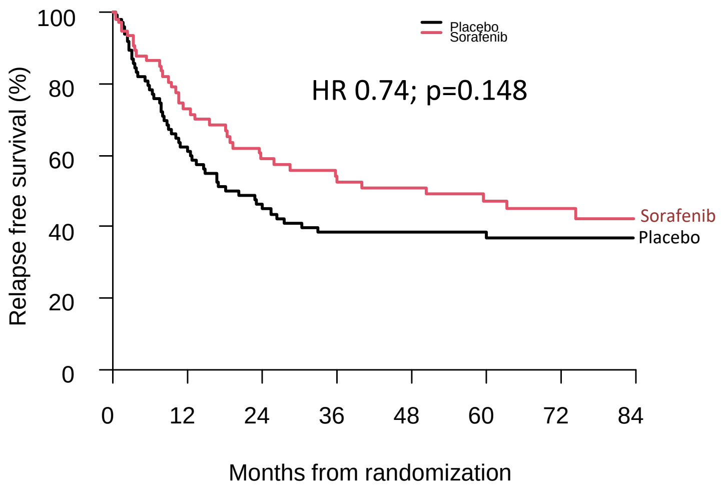

B

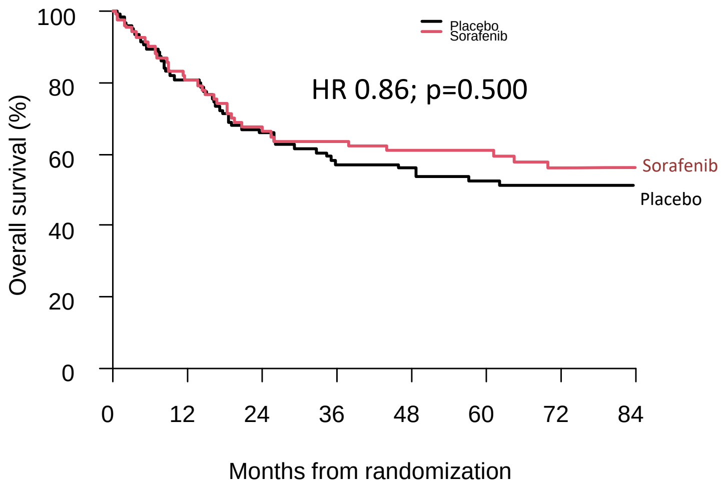

Supplemental Figure SF7. Relapse-free survival (A) and overall survival (B) only for transplanted patients.

A

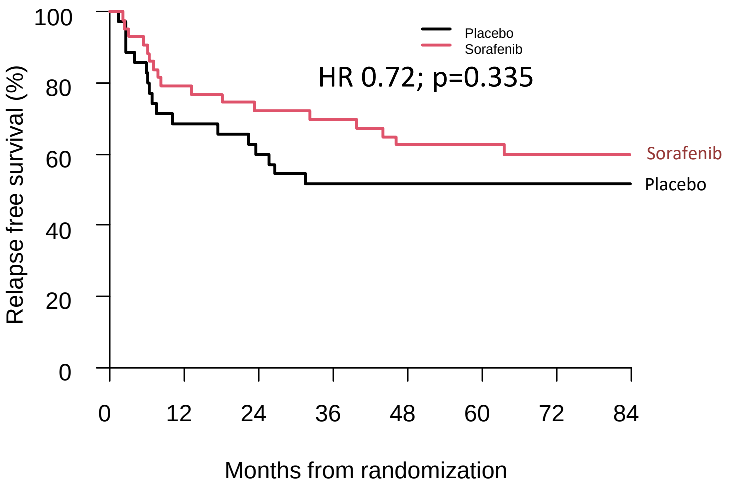

B

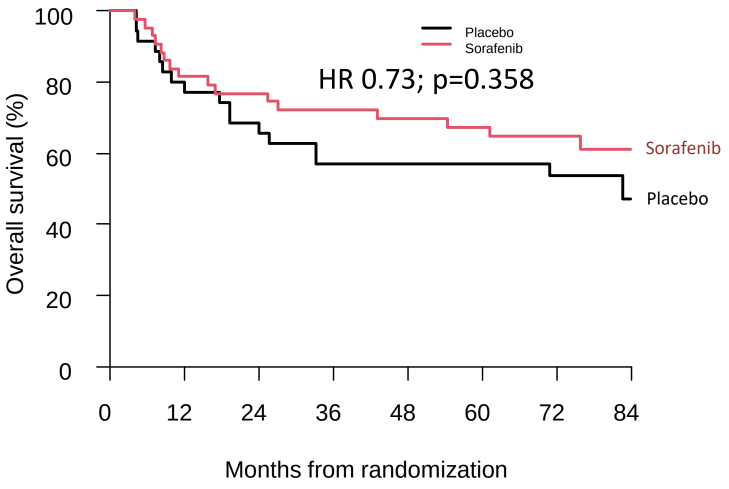

Supplement: Supplementary file 1 — Supplemental Material [file 41375_2021_1148_MOESM1_ESM.pdf]
